# Modulation of alpha and gamma oscillations related to retrospectively orienting attention within working memory

**DOI:** 10.1111/ejn.12589

**Published:** 2014-04-17

**Authors:** Claudia Poch, Pablo Campo, Gareth R Barnes

**Affiliations:** 1 Departamento de Psicología Biológica y de la Salud, Universidad Autónoma de MadridCampus de Cantoblanco, 28049, Madrid, Spain; 2 Department of Basic Psychology, Autonoma University of MadridMadrid, Spain; 3 Wellcome Trust Centre for Neuroimaging, University College LondonLondon, UK

**Keywords:** attention, magnetoencephalography, oscillatory activity, retro-cues, working memory

## Abstract

Selective attention mechanisms allow us to focus on information that is relevant to the current behavior and, equally important, ignore irrelevant information. An influential model proposes that oscillatory neural activity in the alpha band serves as an active functional inhibitory mechanism. Recent studies have shown that, in the same way that attention can be selectively oriented to bias sensory processing in favor of relevant stimuli in perceptual tasks, it is also possible to retrospectively orient attention to internal representations held in working memory. However, these studies have not explored the associated oscillatory phenomena. In the current study, we analysed the patterns of neural oscillatory activity recorded with magnetoencephalography while participants performed a change detection task, in which a spatial retro-cue was presented during the maintenance period, indicating which item or items were relevant for subsequent retrieval. Participants benefited from retro-cues in terms of accuracy and reaction time. Retro-cues also modulated oscillatory activity in the alpha and gamma frequency bands. We observed greater alpha activity in a ventral visual region ipsilateral to the attended hemifield, thus supporting its suppressive role, i.e. a functional disengagement of task-irrelevant regions. Accompanying this modulation, we found an increase in gamma activity contralateral to the attended hemifield, which could reflect attentional orienting and selective processing. These findings suggest that the oscillatory mechanisms underlying attentional orienting to representations held in working memory are similar to those engaged when attention is oriented in the perceptual space.

## Introduction

Even though we are constantly exposed to an environment full of sensory stimuli, we are able to selectively process relevant events most of the time (Asplund *et al*., 2010; Anton-Erxleben & Carrasco, 2013). Selective attention modulates information processing efficiency, allowing us to pick up relevant information and, equally importantly, ignore irrelevant information (Jiang & Chun, 2001; Mevorach *et al*., 2010). Many studies have investigated the mechanisms by which selective attention contributes to the differentiation of relevant from irrelevant information during perception (Posner, 1980; Duncan, 1984; Lu & Dosher, 1998; Luck & Vecera, 2002; Carrasco *et al*., 2004); and others have explored how attention modulates cerebral activity, showing that attended stimuli elicit greater responses than ignored stimuli (Motter, 1993; Luck *et al*., 1997; Corbetta, 1998; Hillyard & Anllo-Vento, 1998) [for a review, see Kastner & Ungerleider (2000)]. However, notwithstanding the great interconnection between attention and working memory (WM) (Awh *et al*., 2000; Oberauer, 2002; Fougnie & Marois, 2006; Chun & Turk-Browne, 2007), very few studies have considered the mechanisms of attentional control over representations held in WM (Gazzaley & Nobre, 2012). Selective attention has been shown to filter what is encoded and maintained in WM (Rutman *et al*., 2010; Zanto *et al*., 2011). Interestingly, differences in filtering efficiency, i.e. the ability to hold critical information while ignoring irrelevant information, has been shown to contribute to individual differences in WM (Vogel *et al*., 2005; Fukuda & Vogel, 2009; Fukuda *et al*., 2010; Jost *et al*., 2011). Whereas these studies have focused on how selective attention mechanisms are engaged for gating the encoding of relevant items (Kuo *et al*., 2011) or relevant features (Poch *et al*., 2010) into WM, several studies have shown that, in the same way that attention can be selectively oriented to bias sensory processing in favor of relevant stimuli in perceptual tasks, it is also possible to retrospectively orient attention to internal representations held in WM (Giffrin & Nobre, 2003; Landman *et al*., 2003; Makovski & Jiang, 2007; Matsukura *et al*., 2007; Makovski *et al*., 2008; Sligte *et al*., 2008). This procedure, referred to as retro-cue, optimises the processing of the contents of WM, reducing the limiting effects related to the restricted capacity of WM (Brady *et al*., 2011; Cowan *et al*., 2012; Fougnie *et al*., 2012). Evidence from neuroimaging studies suggests that the mechanisms for orienting attention during the period after the to-be-remembered stimulus has disappeared are similar to the mechanisms for attentional modulation during perception (Lepsien *et al*., 2005; Lepsien & Nobre, 2006, 2007; Nasr *et al*., 2008; Nobre *et al*., 2008; Dell'Acqua *et al*., 2010; Kuo *et al*., 2011, 2014). If retro-cues trigger top-down biasing mechanisms that operate on representations being stored in WM (Gazzaley & Nobre, 2012), it is reasonable to consider that orienting attention to a specific item or items will be accompanied by keeping the irrelevant items from being further maintained. It has been proposed that oscillatory neural activity in the alpha band serves as an active functional inhibitory mechanism (Lopes da Silva, 1991; Jokisch & Jensen, 2007; Klimesch *et al*., 2007; Jensen & Mazaheri, 2010; Jensen *et al*., 2012; Klimesch, 2012). This notion has been supported by subsequent research showing that orienting attention to one visual hemifield induced a posterior contralateral decrease and/or ipsilateral increase in alpha power, signaling an active facilitative vs. an inhibitory role of alpha oscillatory activity (Worden *et al*., 2000; Siegel *et al*., 2008; Capotosto *et al*., 2009; Rihs *et al*., 2009; Sauseng *et al*., 2009; Snyder & Foxe, 2010; Handel *et al*., 2011; Bauer *et al*., 2012b; Capilla *et al*., 2014). To date, however, no one has explored whether this modulation of alpha oscillations also takes place when attention is directed to specific representations within WM. Additionally, invasive studies in monkeys have shown that attended stimuli induce changes in gamma band activity over the occipital visual cortex (Fries *et al*., 2001), and this modulation of the gamma band activity linked to orienting attention has also been reported in humans (Engel *et al*., 2001; Fan *et al*., 2007; Doesburg *et al*., 2008; Bauer *et al*., 2012b). In light of these observations, it would be also interesting to determine whether the oscillatory patterns in the gamma band associated with orienting attention to external perceptual representations can also be observed when attention is oriented to internal representations in WM.

This aim of this study was to characterise the patterns of neural oscillatory activity when selective attention operates within WM. Accordingly, we measured neural activity recorded with magnetoencephalography (MEG) while participants performed a change detection task, in which a spatial retro-cue was presented during the maintenance period, indicating which item was relevant for subsequent retrieval (Lepsien & Nobre, 2006). Crucially, this condition was compared with other trials in which a non-informative cue (a so-called neutral cue) was presented. If the selective attention processes occurring during WM maintenance are similar to the mechanisms for voluntary attentional orientation during perception (Gazzaley & Nobre, 2012), then it could be hypothesised that information provided by spatial retro-cues will induce the same modulation of neural oscillatory activity in the alpha and gamma bands in response to ignored and attended stimuli observed in the perceptual space. More specifically, considering previous evidence (Jensen *et al*., 2007; Fries *et al*., 2008; Siegel *et al*., 2008; Tiesinga & Buia, 2009; Munneke *et al*., 2012; Kuo *et al*., 2014), we hypothesise that attentional orienting will be reflected in a modulation of oscillatory activity in the visual cortex. Accordingly, we expected that oscillatory activity would be increased ipsilaterally to the processed hemifield in the alpha band, and contralaterally to the processed hemifield in the gamma band in the same regions (Peters *et al*., 2012).

## Materials and methods

### Participants

Seventeen adult subjects [mean age, 25.36 years; standard deviation (SD), 3.13 years; range, 22–32 years; nine females], without any history of neurological or psychiatric illness, volunteered for participation in the study, which was approved by the local ethical committee of the Center of Biomedical Technology, and gave written consent, in accordance with the Declaration of Helsinki, after the nature of the procedures involved had been explained to them. Participants were right-handed according to the Edinburgh Handedness Inventory (Oldfield, 1971).

### Stimuli and tasks

**Figure 1:**
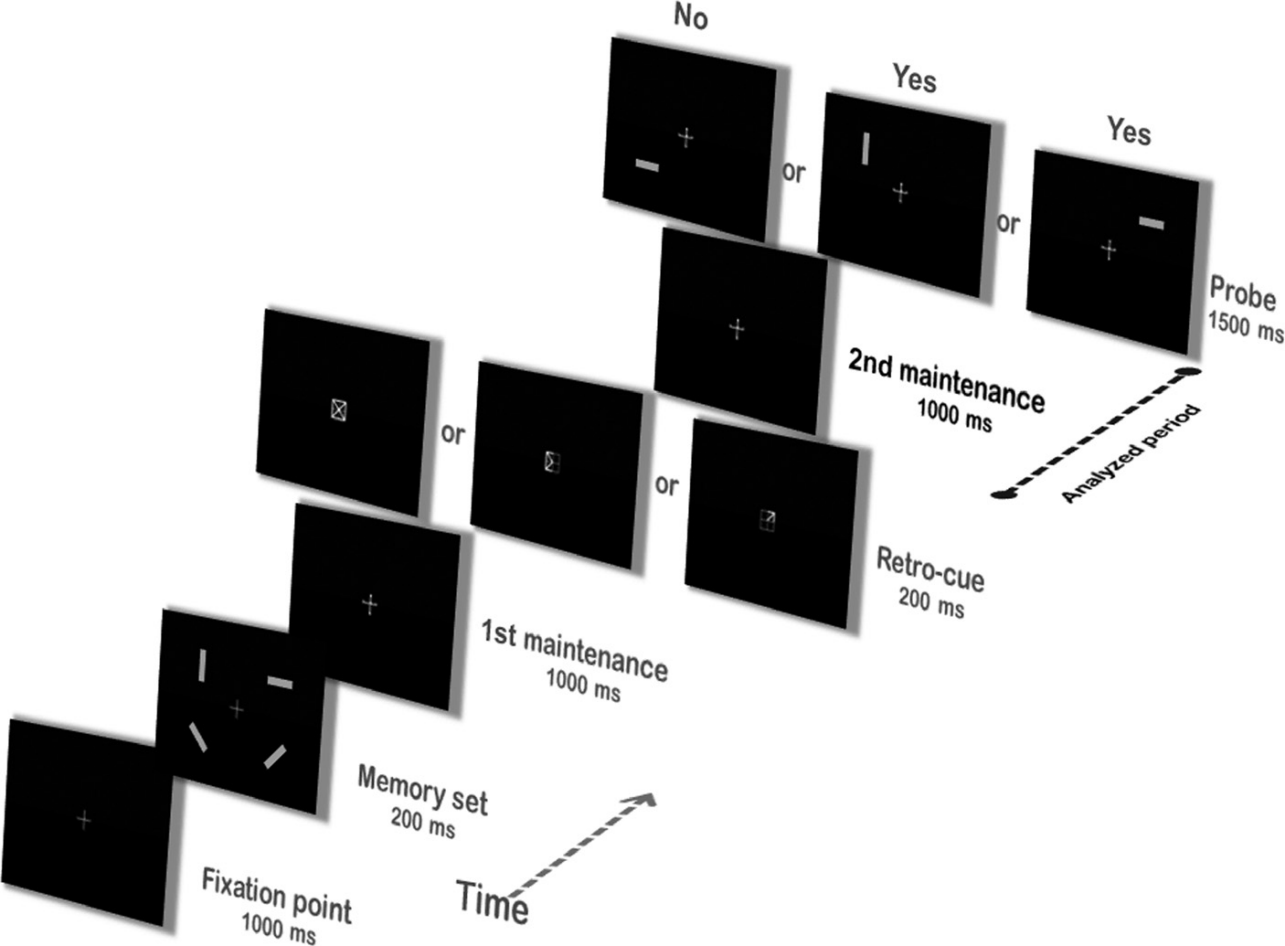
Schematic illustration of the experimental task.

The experimental task was adapted from a retro-cueing task developed by Giffrin and Nobre (Giffrin & Nobre, 2003) [see also Lepsien & Nobre (2006)]. At the start of each trial, participants first saw a 1000-ms white central fixation cross. This was followed by a sample memory set, consisting of four gray rectangles with different orientations displayed in four locations on a black background. The to-be-remembered array remained on the screen for 200 ms, in order to discourage participants from making saccadic eye movements to scan the individual items. After a 1000-ms delay interval, participants could be presented with either an informative spatial cue (i.e. retro-cue) or with no cueing information (i.e. neutral cue). A retro-cue consisted of one or two arrows originating from the fixation cross pointing to one or two of the four locations that had been occupied by a rectangle in the memory array, thus indicating where a relevant item or items were present (validity 100%) (Lepsien *et al*., 2005; Matsukura *et al*., 2007). The neutral cue consisted of four arrows originating from the fixation cross pointing to each of the four locations, thus providing no information regarding the relevant item. Cues were presented for 200 ms, and were followed by another 1000-ms delay interval. Finally, participants were presented with a single rectangle in one location for 1500 ms, during which they were required to respond. The task was to indicate, by button press, whether the probe was present or absent in the to-be-remembered array. Following this response period, a blank screen was shown for 1800 ms before the onset of the next trial (Fig.1). A total of 360 trials were presented, of which 120 had retro-cues indicating one location, 120 had retro-cues indicating two locations, and 120 had neutral cues. Cues pointing to one or two locations were used in order to determine whether attention can be reoriented within WM to more than one item, such as in the perceptual space (Awh & Pashler, 2000; Makovski & Jiang, 2007). The experiment lasted for ∼40 min, and was performed in one single session during MEG scanning.

### Data acquisition and analysis

#### MEG recordings and preprocessing

MEG data were obtained with a whole-head 306-channel Vector-view system (Elekta-Neuromag, Helsinki, Finland), consisting of 102 magnetometers and 204 orthogonal planar gradiometers. The signal was recorded continuously at a sampling rate of 600 Hz with an online bandpass filter from 0.1 to 200 Hz. The head position relative to the sensor array was measured at the beginning of the session with four head position indicator coils. Prior to the recording session, the anatomical landmarks (nasion and auricular) and extra points of the head shape were obtained with a 3D digitiser (Fastrak Polhemus, Colchester, VA, USA).

Visually detectable bad channels were removed prior to signal preprocessing. External noise was removed with the signal space separation method implemented with max filter software (Taulu *et al*., 2004). Data analyses were conducted with the 102 magnetometer channels. Further analyses were performed with spm8 (http://www.fil.ion.ucl.ac.uk/spm/). Data were first downsampled to 300 Hz and filtered with a high cutoff of 150 Hz, and then epoched offline to obtain 1700-ms data segments corresponding to 500 ms of baseline and 1200 ms after the retro-cue. We analysed epoched data during this period for each trial, for each condition, for each participant. Trials were visually inspected, and rejected when they contained sensor or muscular artefacts, and/or eye blinks.

#### Time–frequency (TF) analysis

The TF decomposition was performed with a continuous Morlet wavelet transform (Mallat, 1998), from 4 to 80 Hz in 1-Hz steps, with a relation *f*_0_/σ_*f*_, where σ_*f*_ = 1/(2πσ_*t*_) set to 7. Each epoch was baseline-corrected {*P*_corrected_(*t, f*) = [*P(t, f*) − *P*_baseline_(*f*)]/*P*_baseline_(*f*)}, and then averaged to obtain the induced activity in each condition. TF datasets were transformed into Neuroimaging Informatics Technology Initiative images. Specifically, the 4D [space (*x*,*y*), time, frequency] datasets were converted into a 3D data (channel space × time). In this case, power was averaged in the alpha (8–14 Hz) and gamma (50–80 Hz) bands to obtain scalp–time images. A second-level 2 × 2 anova with the factors hemifield (left vs. right) and load (one element vs. two elements) was performed.

#### Source analysis

The linearly constrained minimum variance scalar beamformer spatial filter algorithm (Sekihara *et al*., 2004), as implemented in spm8, was used to generate maps of source activity in a 10-mm grid, with the use of a single-shell forward model fit to the inner skull surface of the inverse normalised spm template (Nolte, 2003). Coregistration to Montreal Neurological Institute space was carried out with the three anatomical landmarks and the extra digitalised points. The time-windows for covariance computation (and hence source inversion) windows were chosen on the basis of the sensor-level TF analysis results. For alpha band activity (8–14 Hz), a covariance window of 1000 ms after the presentation of the retro-cue was used. For gamma band activity (50–80 Hz), a window of 400 ms after the presentation of the retro-cue was used. Summary statistics images were calculated by subtracting the neutral condition image from the active conditions, generating four volumetric images for each participant. A second-level 2 × 2 anova with the factors hemifield (left vs. right) and load (one element vs. two elements) was performed.

#### Statistics

For both source and sensor-level analysis, we corrected for multiple comparisons by using Gaussian random field theory (Worsley *et al*., 1996; Kiebel & Friston, 2004a,b2004b), as implemented in spm8. At the sensor level, we produced maps of band-limited power (alpha or gamma) over time and space (at each sensor), and computed either peak-level or cluster-level significance. At the source level, we collapsed the data over time to produce volumetric images of power change (for the alpha and gamma bands), which, over space, were corrected at either the peak or the cluster level. In the case of cluster-level tests, we used a cluster-defining threshold of *P* < 0.001 (uncorrected). At the source level, we were able to bring to bear our prior hypotheses of contralateral visual cortex gamma power change, and therefore used a 2.5-cm sphere centered at the peak of ipsilateral alpha enhancement as our *a priori* region of interest.

## Results

### Behavioral performance: retro-cue task

In order to evaluate differences across conditions, we performed a repeated measures anova with the within-factor condition (one element, two elements, and neutral) for the parameters accuracy and reaction time.

### Accuracy

Analysis of task accuracy was performed after *d*′ values had been estimated for each condition (Abdi, 2007). Analysis revealed a main effect of condition (*F*_2,32_ = 23.99, *P* < 0.001, η^2^ = 0.60). Planned comparisons showed that participants were more accurate in the one-element condition (mean = 0.89, SD = 0.16) than in the two-element condition (mean = 0.78, SD = 0.16) (*t* = 5.05, *P* < 0.001) and the neutral condition (mean = 0.72; SD = 0.15) (*t* = 6.8, *P* < 0.001). We also observed a trend for a better performance in the two-element condition than in the neutral condition (*t* = 2.09, *P* = 0.053).

### Reaction time

There was a main effect of stimulus condition (*F*_2,32_ = 234.83, *P* < 0.001, η^2^ = 0.94), revealing that participants' reaction time increased with increasing number of to-be-attended items. Participants were slower in the neutral condition (mean = 509.89 ms, SD = 58.45 ms) than in both the one-element condition (mean = 386.05 ms, SD = 51 ms) (*t*_16_ = 17.07, *P* < 0.001) and the two-element condition (mean = 484.43 ms, SD = 58.85 ms) (*t*_16_ = 17.14, *P* < 0.001). Participants were also slower in the two-element condition than in the one-element condition (*t*_16_ = 5.23, *P* < 0.001).

### Neuroimaging results

#### Sensor space analysis

TF statistical analysis was performed on baseline normalised data. Using this analysis, we tested for average effects of condition, i.e. effects that were significant across all experimental conditions relative to the pre-stimulus baseline. Importantly, this contrast was orthogonal to the main effects of interest, and was used only to define a TF window of interest.

**Figure 2:**
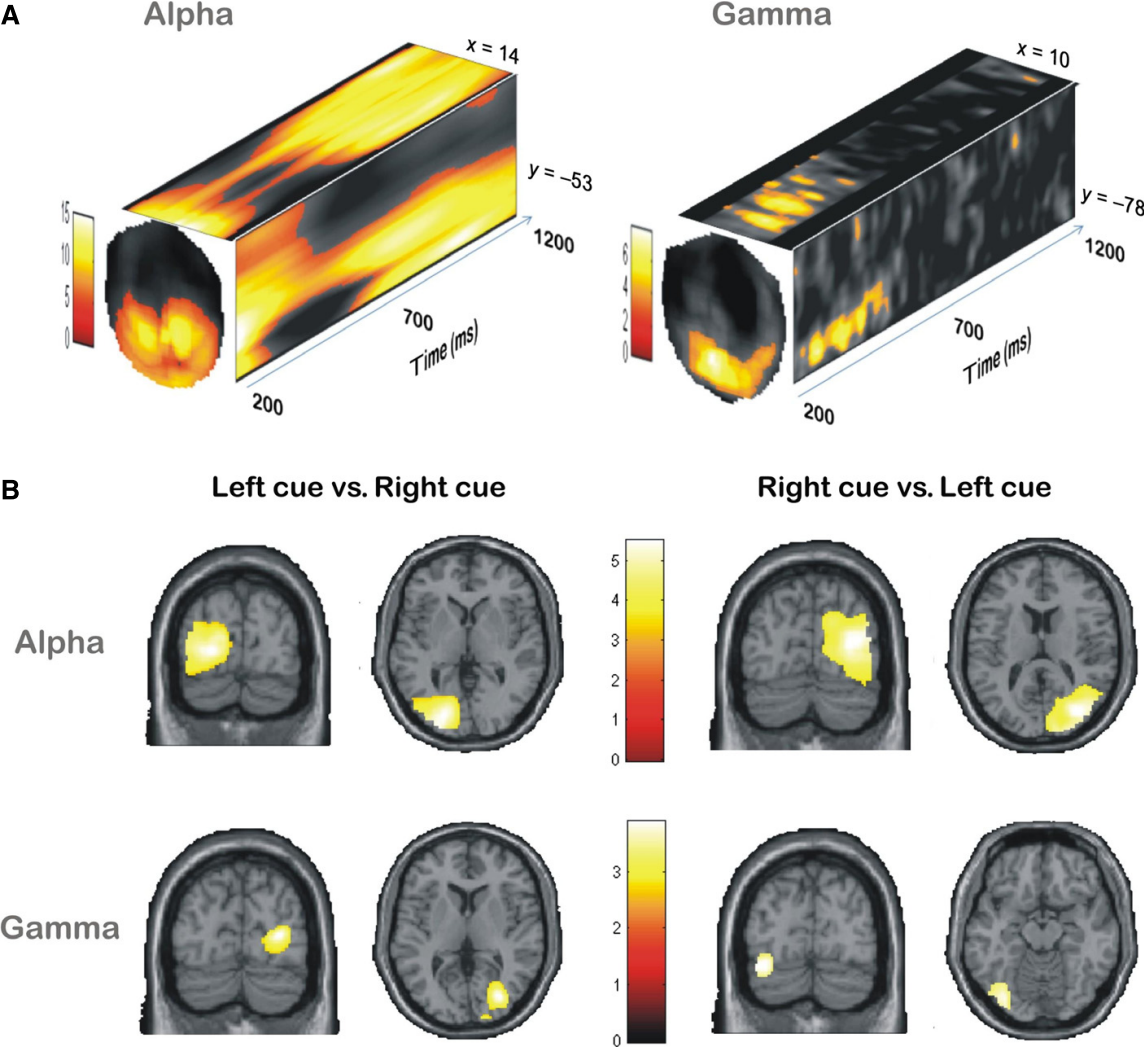
(A) *t*-Statistic sensor-level maps of significant activity in experimental conditions relative to baseline (between 500 and 0 ms before stimulus onset) in the alpha (*P* < 0.05, FEW-corrected) and gamma (*P* < 0.05, FEW-corrected at the cluster level) frequency bands as a function of time. The front faces of the cubes show the distribution over the sensors of significant alpha (left) and gamma (right) band power changes at time bins of 700 and 550 ms, respectively. The top and side faces of these cubes show the projection of the maxima within these individual *t*-statistic maps onto the outer cube surface over time. (B) Localisation of significant cortical sources for alpha and gamma resulting from comparison of the left retro-cue condition with the right retro-cue condition (left column), and comparison of the right retro-cue condition with the left retro-cue condition (right column). For display purposes, only the *t*-value images are thresholded at *P* < 0.001 and *P* < 0.005, uncorrected, for alpha and gamma bands, respectively. The color scales indicate *t*-values.

To illustrate the oscillatory components present during orientation, averaged frequency responses were computed for each sensor, with the use of all trials for each participant for frequencies from 8 to 14 Hz. We found a significant [*P* < 0.05, familywise error (FEW)-corrected over sensors and time] sustained increase in alpha power after retro-cue presentation (i.e. 200–1200 ms) over posterior sensors (Fig.2A).

We also evaluated whether neural activity in the gamma frequency band showed an orienting effect. A significant gamma power increase (*P* < 0.05, FEW-corrected at the cluster level over sensors and time) occurred during the retro-cue delay period over posterior sensors (Fig.2A). The interval where this activity was significant was between 250 and 660 ms after the retro-cue presentation.

#### Source space analysis

Having established a significant modulation of neural activity in the alpha and gamma bands at the level of MEG sensors, we were interested in determining the underlying generative sources. Oscillatory activity was reconstructed on the basis of the time-windows where significant modulation of neural activity was found at the sensor level (see above).

An ipsilateral increase in alpha activity was found for the right condition in the right occipital cortex (*P* < 0.05, whole-brain FEW-corrected) as compared with the left condition. Likewise, an increase in the left occipital cortex was observed for the left condition (*P* < 0.05, whole-brain FEW-corrected) as compared with the right condition (Fig.2B).

On the basis of our prior hypotheses of contralateral gamma power increases at ipsilateral alpha enhancement sites, we tested within *a priori*-defined 20-mm radius volumes centered at *x* = 32, *y* = −76 and *z* = 14 in the right hemisphere, and at *x* = −22, *y* = 84 and *z* = 2 in the left hemisphere. A contralateral increase in gamma power was observed in the right occipital cortex when participants were cued to orient attention to items in the left hemifield (*P* < 0.05, FEW-corrected). Similarly, gamma activity was increased in the left occipital cortex when participants were cued to orient attention to items in the right hemifield (*P* < 0.05, FEW-corrected) (Fig.2B). We did try this analysis without the use of a region of interest, but found no FEW-corrected significant effect at the whole-brain level.

We did not find a load-dependent modulation of oscillatory activity in either the alpha or gamma frequency bands.

## Discussion

Previous studies have shown that orienting of attention involves modulation of oscillatory activity, mainly in posterior sensory cortices. Such studies have demonstrated that, when attention is directed to one hemifield, with tasks that vary in stimulus modality, there is not only an increase in alpha activity in the hemisphere ipsilateral to the attended hemifield, but also a decrease in the contralateral one (Worden *et al*., 2000; Thut *et al*., 2006; Freunberger *et al*., 2008; Siegel *et al*., 2008; Rihs *et al*., 2009; Sauseng *et al*., 2009; van Dijk *et al*., 2010; Haegens *et al*., 2010; Huang & Sekuler, 2010; Snyder & Foxe, 2010; van Ede *et al*., 2011; Grent-'t-Jong *et al*., 2011; Handel *et al*., 2011; Bauer *et al*., 2012a,b2012b; Capilla *et al*., 2014). These findings led to the hypothesis that alpha band oscillations play an active role in information processing by inhibiting task-irrelevant areas (Lopes da Silva, 1991; Foxe *et al*., 1998; Klimesch *et al*., 2007; Jensen & Mazaheri, 2010; Jensen *et al*., 2012; Klimesch, 2012); specifically, the unattended visual stream is associated with strong alpha oscillations (Jensen *et al*., 2012). The results presented in this article are compatible with the existence of a similar oscillatory neural mechanism when attention is oriented to representations held in WM and those observed when orienting attention in the perceptual space. We found that, when participants deployed attention to representations maintained in WM, there was an increase in alpha power ipsilateral to the attended hemifield, and/or a decrease in contralateral sensors. This modulatory effect was localised to ventral occipital cortices (Fig.2B). This finding is similar to those of a recent study using a change detection paradigm with a retro-cue condition (Sligte *et al*., 2009). As the improvement in task performance has been interpreted as reflecting the protection of behaviorally relevant information from inter-item competition (Murray *et al*., 2013), such an ipsilateral alpha increase appears to support its suppressive role (Jensen & Mazaheri, 2010), i.e. a functional disengagement of task-irrelevant regions (van Dijk *et al*., 2010; Haegens *et al*., 2010). Whereas this modulatory effect of alpha amplitude has been previously related to suppression of irrelevant parts of the visual field (Worden *et al*., 2000; Freunberger *et al*., 2008; Huang & Sekuler, 2010; Capilla *et al*., 2014), the current findings demonstrate that this modulation also indicates the suppression of irrelevant items already maintained within WM [see also Snyder & Foxe (2010)]. Interestingly, the modulation of alpha oscillatory activity was accompanied by an increase in gamma band activity in contralateral occipital sites (Buffalo *et al*., 2011; Jensen *et al*., 2012), which has been associated with the deployment of attention to a given visual field (Fries *et al*., 2001; Taylor *et al*., 2005; Womelsdorf *et al*., 2006; Gregoriou *et al*., 2009; Schroeder & Lakatos, 2009; Bosman *et al*., 2012), Therefore, the current findings suggest that modulations of the amplitude of alpha and gamma oscillatory activity in the visual system underpinning the allocation of attentional resources observed in the perceptual space (Jensen *et al*., 2012; Klimesch, 2012) are also observed during attentional orienting within the representational space. These data are consistent with the idea that ipsilateral alpha enhancement is a protective function that suppresses the processing of irrelevant/distracting information, and that, in order to be effective, needs to be sustained until probe presentation (Bonnefond & Jensen, 2013; Capilla *et al*., 2014). Conversely, the shorter contralateral gamma enhancement could be interpreted as a process signaling the deployment of attention to and selection of the task-relevant item related to a location-specific stage of processing, associated with P3b (Andersen *et al*., 2010), which does not required sustained attention (Doesburg *et al*., 2008; Tallon-Baudry, 2009; Tiesinga & Buia, 2009; Rerko *et al*., 2014).

Behaviorally, the current findings strengthen the view that directing attention within WM improves performance in terms of accuracy and response times (Landman *et al*., 2003; Matsukura *et al*., 2007; Makovski *et al*., 2008; Sligte *et al*., 2008; Murray *et al*., 2013). If we consider the modulation of alpha activity as a neural correlate of functional inhibition of task-irrelevant information (Klimesch *et al*., 2007; Jensen & Mazaheri, 2010), then the benefit provided by the retro-cues will rely on the protection of the representation of the cued item from interference by other memory items (Landman *et al*., 2003; Makovski & Jiang, 2007; Matsukura *et al*., 2007). Thus, focusing attention to the cued item will increase the proportion of resources allocated to it (Bays & Husain, 2008), which can solidify those representations (Makovski & Jiang, 2007; Makovski *et al*., 2008). Additionally, we have also shown that this beneficial effect can be observed even when the subset of cued items exceeds one (Makovski & Jiang, 2007), although performance was worse than when only one item was focused on (Anderson *et al*., 2013). Load-dependent changes in neural activity have been reported during selective attention and WM, reflecting either the amount of irrelevant information that has to be suppressed (Sauseng *et al*., 2009; Jensen *et al*., 2012), or the processing of an increased amount of relevant information (Sauseng *et al*., 2009; Ester *et al*., 2012). However, although we expected to see load-dependent modulation of oscillatory activity, no significant effects of load were observed at either the sensor or the source level.

In summary, we investigated the oscillatory neural mechanisms underpinning attentional orienting within WM. In line with an influential model proposing that alpha band oscillatory activity is increased within cortical regions expected to process irrelevant information, thus serving as an active functional inhibitory mechanism (Jensen & Mazaheri, 2010; Klimesch, 2012), we observed greater alpha activity in a ventral visual region ipsilateral to the attended hemifield. This modulation was accompanied by an increase in gamma activity contralateral to the attended hemifield (Womelsdorf & Fries, 2007; Fries, 2009; Jensen *et al*., 2012). Thus, the current findings suggest that the oscillatory mechanisms underlying attentional orienting to representations held in WM are similar to those observed when attention is oriented in the perceptual space.

## Abbreviations

FEW,: familywise error
MEG,: magnetoencephalography
SD,: standard deviation
TF,: time–frequency
WM,: working memory
